# Postsynthetic functionalization of covalent organic frameworks

**DOI:** 10.1093/nsr/nwz122

**Published:** 2019-08-20

**Authors:** Yusran Yusran, Xinyu Guan, Hui Li, Qianrong Fang, Shilun Qiu

**Affiliations:** State Key Laboratory of Inorganic Synthesis and Preparative Chemistry, Departement of Chemistry, Jilin University, Changchun 130012, China

**Keywords:** covalent organic frameworks, crystalline materials, porous materials, postsynthetic functionalization

## Abstract

Covalent organic frameworks (COFs) have been at the forefront of porous-material research in recent years. With predictable structural compositions and controllable functionalities, the structures and properties of COFs could be controlled to achieve targeted materials. On the other hand, the predesigned structure of COFs allows fruitful postsynthetic modifications to introduce new properties and functions. In this review, the postsynthetic functionalizations of COFs are discussed and their impacts towards structural qualities and performances are comparatively elaborated on. The functionalization involves the formation of specific interactions (covalent or coordination/ionic bonds) and chemical reactions (oxidation/reduction reaction) with pendant groups, skeleton and reactive linkages of COFs. The chemical stability and performance of COFs including catalytic activity, storage, sorption and opto-electronic properties might be enhanced by specific postsynthetic functionalization. The generality of these strategies in terms of chemical reactions and the range of suitable COFs places them as a pivotal role for the development of COF-based smart materials.

## Introduction

Inspired by the highly complex, yet systematic, organization of porous-nature materials, plentiful efforts have been devoted by researchers to mimicking their properties. Over the past several decades, the regular arrangement of secondary building units of porous inorganic zeolites to establish defined micropores has shown tremendous advantages in high-demand applications [1–4]. On the other hand, with the manageable and controllable porosity under the reticular chemistry principle, metal-organic frameworks (MOFs) have come onto the scene as the second generation of crystalline porous materials [5–7]. In 2005, Yaghi *et al.* designed a new type of crystalline porous polymer by reticulating predesigned organic building blocks into certain 2D or 3D networks, termed covalent organic frameworks (COFs) [8]. This great discovery tackles the bottleneck of the preconception in which covalent linking of organic molecules into crystalline solids is challenging, since it typically affords amorphous solids or disordered materials.

As an emerging class of highly porous organic polymers, COFs have been researched for over a decade. The rigid backbone and precise arrangement of building blocks in COFs afford highly crystalline materials, making them more robust than amorphous organic polymers [9]. From 2005 until the present, the library of COF compounds has been expanded rapidly with the highly enthusiastic interest of researchers. Since COFs are composed solely of light-organic elements (B, C, O, N and S), they have been remarked as having the lowest density among porous materials [10]. This could promise better performance in several applications, especially in gas storage and other highly demanding applications.

The synthesis of COFs allows the formation of periodic skeleton and defined pores [8]. Under a reversible condensation reaction, the classical crystallization issue found in amorphous organic polymers has been addressed fruitfully in COF synthesis provided by a defect-healing mechanism. In principle, there are two distinct components in COF construction, namely linkers (building blocks) and linkages (bonds connecting the building blocks). The myriad availability of building blocks with different sizes and shapes garners flexibility in designing the porosity and topologies of COFs. Nevertheless, the strong covalent linkages contribute to the high chemical and thermal stabilities of COFs, which are crucial aspects in MOFs and inorganic zeolites. Furthermore, the bottom-up synthesis of COFs allows the designable structural framework that provides the control in the structural–property relationship of the resultant product. This encompasses the pore size, surface physicochemical environment and structural modification. The flexible pore wall decoration (the incorporation of pendant groups and/or active sites) in COFs offers potential exploration in structural functionalization.

**Scheme 1.**
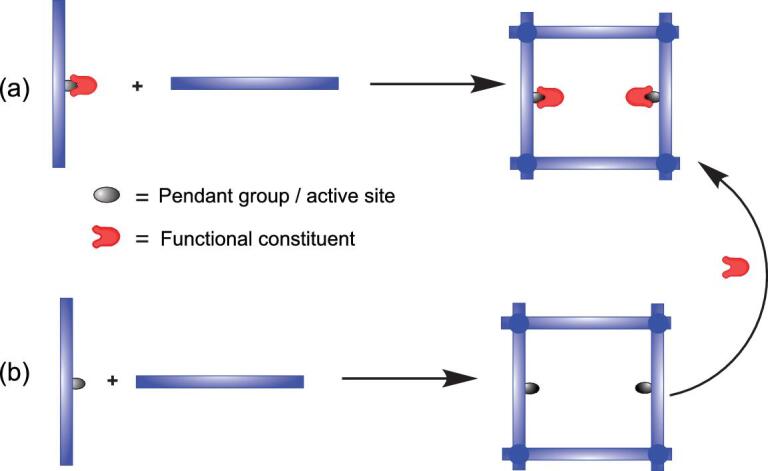
Structural functionalization approaches in COFs. (a) Bottom-up approach and (b) postsynthetic functionalization approach.

Structural functionalization in COFs provides structural qualities and performance enhancements, including gaseous/adsorbate uptake–release, optical/electrical responses and host–guest interactions. Generally, the structural functionalization of COFs can be performed by pre-designing the building blocks (bottom-up approach) and by the postsynthetic functionalization approach. In the bottom-up approach, the building blocks are previously modified with the desired functional groups that are stable during COF synthesis. Meanwhile, in the postsynthetic approach, the functional groups are introduced or attached to the pore surface of COFs while maintaining the structural integrity. Comparatively, the later approach is technically more viable compared to the former one. Indeed, several reported works have shown the structural and performance enhancement of COFs through postsynthetic functionalization over a wide range of applications. In this review, we summarize the postsynthetic functionalization of COFs that encompasses the functionalization of the pendant groups and the linkages, involving the formation of specific bonds (covalent and coordination/ionic bonds), chemical reaction (oxidation/reduction reaction) and host–guest interaction. In addition, we further elaborate comparatively on the contribution of this functionalization to the structural qualities and performances of the resultant COFs.

## Structural functionalization approaches in COFs

The design flexibility of COFs through the wide selectivity of building blocks offers opportunities to decorate their backbone with functional moieties. Meanwhile, the adjustable pore size and environment of COFs make them an ideal scaffold for the incorporation of new functional groups. Technically, structural functionalization of COFs can be divided into two approaches: bottom-up and postsynthetic approaches, which are illustrated in Scheme 1. Both approaches produce the desired functionalized COFs with different properties in terms of crystallinity, porosity and stability.

**Table 1.** Typical reactive pendant groups appended on COF backbone useful for further functionalization

Pendant groups	Coupling agents	Bond formed
-N_3_ (azide)	-C≡C- (alkyne)	Triazole
-OH (hydroxyl)	-C(O)-O-C(O)- (anhydride)-NCS (isothiocyanate)-Br (halide)	EsterThiorcarbamateEther
-NH_2_ (amine)	-C(O)-O-C(O)- (anhydride)	Amide
-≡N (nitrile)	-NH_2_ (amine)	Amidoxime
-SH (thiol)	-C=C- (alkene)	Thioether

## Bottom-up functionalization

In the bottom-up functionalization approach, the functional moieties of COFs are originated from the predesigned building blocks (Scheme 1a). One representative piece of work on this approach was reported by Wang *et al.*, in which they synthesized 2D mesoporous COFs with chiral functional groups (COF-LZU-72 and COF-LZU-76) [11]. To produce those COFs, the basic building block (diamino-*p*–terphenyl) was modified with chiral pyrrolidine moiety previously to afford the desired functionalized unit. However, it should be noted that a certain protective group might be needed during the pre-modification. A similar vision was then adopted to generate several functionalized COFs including thiol-functionalized 2D COF (Thio-COF) and sulfonic acid-functionalized 2D COF (TpPa-SO_3_H) [12,13].

However, besides the probable protection–deprotection process, there are other crucial disadvantages based on the bottom-up approach, such as the difficulties in synthesizing functionalized building blocks and challenges in the incorporation of large or bulky constituents that may disturb the regularity of the desired COFs. Thus, this approach seems hard for a variety of functional groups.

## Postsynthetic functionalization

While some functionalized COFs can be obtained via a bottom-up approach, a postsynthetic functionalization strategy provides a more robust platform (Scheme 1b). Comparatively, it offers a versatile avenue to introduce wider types of functional moieties into COFs without altering the structural regularity. More importantly, it avoids or minimizes the involvement of undesired side reactions during reticulation and functional incorporation. The functionalization of COFs via a postsynthetic approach involves specific bond formation, chemical reactions and host–guest interactions between the pendant groups or the active sites of the established COF and functional constituents. On this point, we classify them into three categories: pendant group and skeleton functionalization, linkage functionalization and host–guest functionalization.

### Pendant groups and skeleton functionalization

The specific pendant groups (Table 1) and the skeleton of COFs can be sufficiently functionalized by treating with desired functional constituents. Particularly, the interaction between them includes either the formation of covalent or ionic/coordination bonds or oxidation/reduction reactions, depending on the reaction conditions and types of functional modifiers. In the following sub-section, both interactions are elaborated on, along with their contribution to the stability and performance.

**Figure 1.**
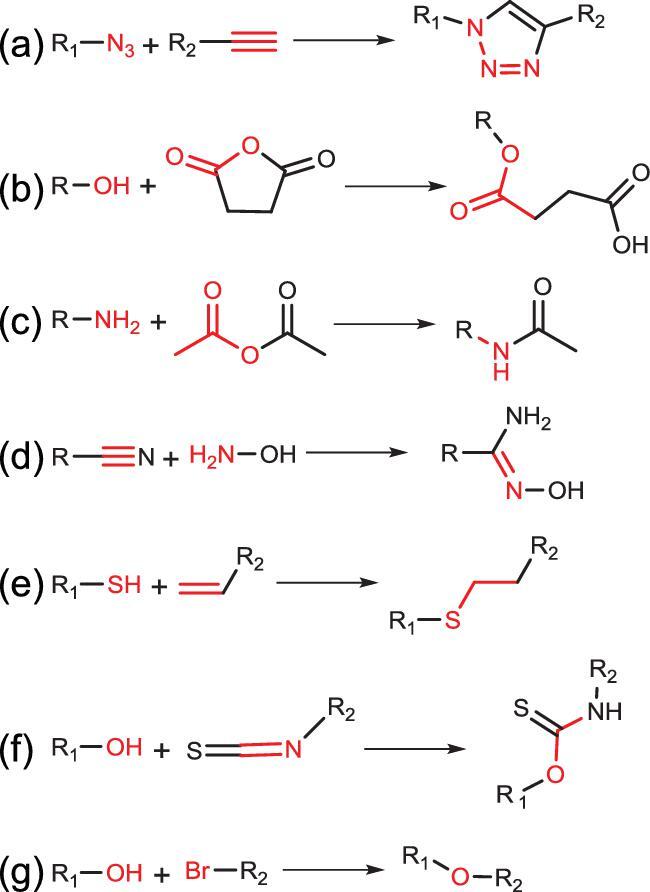
Formation of typical covalent bonds adopted in postsynthetically functionalizing the structural backbone of COFs. (a) Triazole, (b) ester, (c) amide, (d) amidoxime, (e) thioether, (f) thiocarbamate and (g) ether.

**Figure 2.**
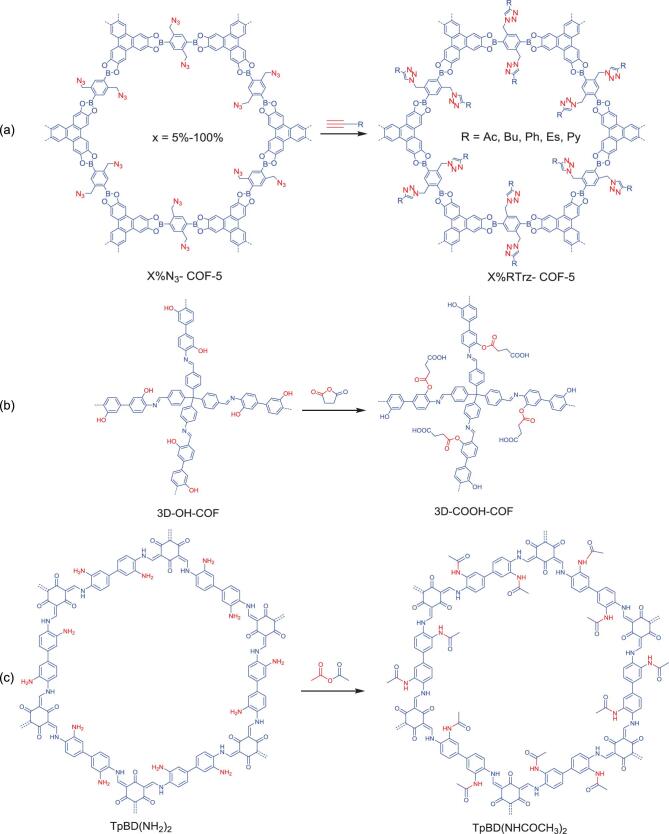
Postsynthetic functionalization of pendant groups of COFs via various covalent bonds. (a) Functionalization of azide-appended X%N_3_-COF-5 series into X%RTrz-COF-5 series via triazole bond. (b) Functionalization of hydroxyl-appended 3D-OH-COF into 3D-COOH-COF via ester bond. (c) Functionalization of amine-appended TpBD(NH_2_)_2_ COF into TpBD(NHCOCH_3_)_2_ via amide bond.

#### Covalent bond

The functionalization of pendant groups of COFs via covalent bonds is performed by linking the incoming functional constituents with the available pendant groups on the pore channel. Practically, typical COFs with reactive pendant groups are designed as the scaffold. Table 1 summarizes the typical pendant groups available in COFs and their corresponding coupling agents that have been demonstrated in functionalizing COFs. The typical bonds of triazole, ester, amide, amidoxime, thioether, thiocarbamate and ether have been adopted so far to postsynthetically functionalize COF as illustrated in Fig. 1.

The triazole bond that can be prepared under a CuI-assisted click reaction between azide (R-N_3_) and ethynyl (R≡CH) groups has been widely employed to functionalize COFs (Fig. 1a). For example, treating azide-appended COFs (X%N_3_-COF-5) with alkynes afforded triazole-functionalized COF-5 (X%RTrz-COF-5) (Fig. 2a) [14]. Notably, this functionalization was compatible for various functional groups attached on the ethynyl groups (R = acetyl (Ac), butyl (Bu), benzyl (Ph), methyl ester (Es) or pyrene (Py)). In addition, it preserved the crystalline nature of the former scaffold and was able to control the density of the triazole groups in the final product. The resultant X%RTrz-COF-5 series possessed different affinities to the different gases and displayed varied adsorption capabilities, which is a potential for

gas separation. Meanwhile, an extended chiral centre and catalytic active site has successfully been endowed within [HC≡C]_x_-TPB-DMTP-COFs by reacting alkynyl (-C≡CH) pendant groups with azide-containing pyrrolidine (N_3_-Py) [15]. The resultant [(*S*)-Py]_x_-TPB-DMTP-COFs contained chiral and catalytic active pyrrolidine moieties. Thus, they have potential as a chiral organocatalyst. Remarkably, [(*S*)-Py]_x_-TPB-DMTP-COFs were crystalline with a significant porosity intake. As a heterogeneous chiral organocatalyst, [(*S*)-Py]_x_-TPB-DMTP-COFs could catalyse the organic Michael-addition reaction—a basic C–C bond-formation reaction for synthesizing various important synthetics and natural products [16]. In addition, [(*S*)-Py]_x_-TBP-DMTP-COFs were able to promote asymmetric C–C bond formation in water under ambient temperature with prominent activity, enantioselectivity, recyclability and environmental benignity compared to the other metal-based catalysts. For instance, [(*S*)-Py]_0.17_-TBP-DMTP-COF proceeded smoothly and cleanly to C–C bond formation with 100% conversion in 12 h (*e.e.* = 92% and *d.r. =* 90/10), which largely exceeded the molecular (*S*)-Py catalyst. Furthermore, several other COFs have been functionalized via triazole linkage, including the transformation from [CH≡C]_x_-H_2_P-COF into [Pyr]_x_-H_2_P-COF and [CH≡C]_0.5_-TPB-DMTP-COF into ([uracil]_0.5_-TPB-DMTP-COF) with preservation of crystallinity and enhanced performance for catalyst and adsorbent applications, respectively [17,18]. Meanwhile, a large molecule of bucky-ball (C_60_) has also been anchored on the pore surface of predesigned X%[N_3_]-ZnPc-COF to afford donor–acceptor heterojunction-based [C_60_]_y_-ZnPc-COF via covalent triazole linkage [19]. As predicted, the resultant [C_60_]_y_-ZnPc-COF exhibited a photoelectric phenomenon with segregated donor–acceptor alignment in which electron-accepting bucky-balls were spatially confined within the nanochannel via triazole units. In addition, it performed photo-induced electron transfer facilitated by an electron-donating COF scaffold and electron-accepting bucky-ball molecules. COFs with high energy-storage performance were achieved by the covalent introduction of redox-active species [20]. In another piece of work, the CO_2_-capture and -separation properties of COFs were tuned via anchoring diverse functional groups from hydrophobic to hydrophilic and from acid to basic through such approaches [21].

Ester bonds have judiciously been applied to functionalize COFs, which were efficiently obtained under a mild and metal-free ring-opening reaction between anhydride and hydroxyl (-OH) groups (Fig. 1b) [22]. Hydroxyl-decorated square-like 2D porphyrin COFs ([HO]_x%_-H_2_P-COFs) were synthesized and employed as scaffolds to demonstrate the structural functionalization of COF via an ester bond [23]. The reaction of these COFs with succinic anhydride generated functionalized COFs with free-carboxyl groups linked by an ester bond ([HO_2_C]_x%_-H_2_P-COFs). This functionalization allows structural rigidity preservation with a well-organized functional constituent within the pore channel and substantial porosity intake. At the end, [HO_2_C]_x%_-H_2_P-COFs were employed as solid sorbents for CO_2_ storage and separation. Remarkably, the [HO_2_C]_x%_-H_2_P-COF sample demonstrated dramatic CO_2_-uptake enhancement relative to [HO]_x%_-H_2_P-COF. In addition, the ideal absorbed solution theory (IAST) calculation was performed based on their CO_2_ and N_2_ sorption isotherms, revealing high CO_2_-absorption selectivity (*S_ads_*) with 323 for [HOOC]_100%_-H_2_P-COF (at 0.1 kPa)—much greater than [HO]_100%_-H_2_P-COF (only 18). Indeed, this performance was much higher than for other reported adsorbents. On another occasion, a predesigned hydroxyl-appended 3D COF (3D-OH-COF) was prepared and further functionalized via an ester bond (Fig. 2b) [24]. A transformation from hydroxyl pendant groups to ester bonds via the ring-opening reaction with succinic anhydride afforded carboxyl-functionalized 3D COF (3D-COOH-COF). With retained crystallinity and sufficient porosity, the resultant 3D-COOH-COF was employed as a selective adsorbent for Nd^3+^ over Sr^2+^ and Fe^3+^ in aqueous solution. Interestingly, Nd^3+^ had the highest uptake at low concentrations, as a result of stronger binding with the framework, while Fe^3+^ possessed the highest uptake at saturation owing to its smaller size. Meanwhile, further IAST calculation showed that 3D-COOH-COF selectively absorbed Nd^3+^ ion over Sr^2+^ ion (with IAST selectivity = 27) and Fe^3+^ ions (IAST selectivity = 18) for a solution containing 5% Nd^3+^ ion and 95% Sr^2+^ or Fe^3+^ ions.

Amide bonds (the strong bonds found in protein) can serve as active sites with secondary amine and carbonyl groups. The incorporation of an amide bond within COFs could trigger the formation of highly porous materials with high chemical stability and abundant active sites. The formation of this bond is typically performed under reaction between primary amine (-NH_2_) and carbonyl groups (Fig. 1c). However, it seems a challenge to incorporate functional groups such as aldehydes, boronic acids or anhydrides, since they are prone to reacting with the building blocks or likely disturb the formation of the rigid backbone of COFs. A chemically stable amino-functionalized TpBD(NH_2_)_2_ was designed by reducing its parent nitro-functionalized COF (TpBD(NO_2_)_2_) [25]. The aminolysis reaction [26] between TpBD(NH_2_)_2_ and acetic anhydride manifested the amide bond containing COF (TpBD(NHCOCH_3_)_2_) (Fig. 2c). Notably, the crystalline was still preserved even after sequential transformation of the COF. Furthermore, the benefit from the high chemical stability in TpBD(NHCOCH_3_)_2_ was applied in lactic acid (LA, pH = 2.2, 0.1 M) adsorption. The TpBD(NHCOCH_3_)_2_ sample exhibited moderate LA uptake (4.0 wt%) compared to TpBD(NO_2_)_2_ and TpBD(NH_2_)_2_ (2.5 and 6.6 wt%, respectively). Indeed, these absorption trends were due to the presence of H-bonding and the chemical stability found in amine and amide-functionalized COFs.

**Figure 3.**
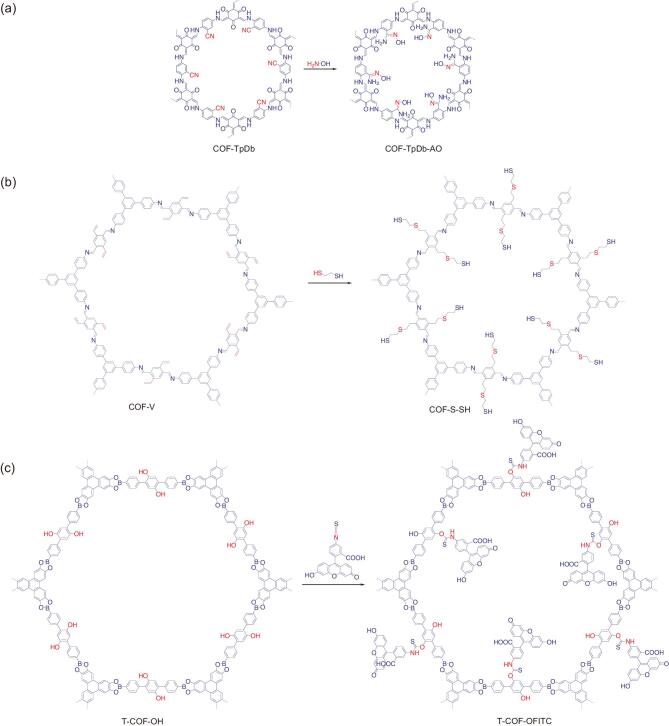
Postsynthetic functionalization of pendant groups of COFs via various covalent bonds. (a) Functionalization of nitrile-appended COF-TpDb into amidoxime-functionalized COF-TpDb-AO via amidoxime bond. (b) Functionalization vinyl-functionalized COF-V into thioether-functionalized COF-S-SH via thioether bond. (c) Functionalization of hydroxyl-appended T-COF-OH COF into T-COF-OFITC via thiocarbamate bond.

In line with the postsynthetic functionalization of COFs mentioned above, another predesigned COF was sequentially functionalized to form a covalent organic nanosheet (CON) with specific functionality [27]. The COF (TpASH) was prepared under a salt-mediated solid-state mixing procedure. Subsequently, TpASH was proceeded into three-sequential postsynthetic functionalization in which its phenolic hydroxyl pendant groups were converted into alkyl-hydroxyl groups under conjugation with glycidol (GIc) in the first step to obtain TpASH-GIc. It was then reacted with 3-aminopropyltriethoxysilane (APTES) to give amine (-NH_2_)-functionalized CONs (TpASH-APTES). In the last step, the folic acid was incorporated into the pore surface of TpASH-APTES CONs to produce the targeted functionalized CONs (TpASH-FA). Interestingly, under sequential functionalization, stacked layers of COFs were delaminated into a few layers of functionalized CONs with enhanced dispersibility while preserving the structural integrity. In the end, the resultant TpASH-FA was employed as a drug carrier for 5-fluorouracyl (5-FU) molecules. Notably, TpASH-FA could load 12% of the 5-FU and established cancer-specific drug release. Indeed, this report signals the future prospect of CONs for drug-delivery application obtained by postsynthetic functionalization. The electron-rich primary amine and anthracene imine are promising active sites for sorption application. The amidoxime bond particularly possesses primary amine and anthracene imine sites. Functionalization of COFs via amidoxime bonds was performed to incorporate those sites that have potential for the generation of adsorbents for nuclear-waste mitigation (Fig. 1d). Cyan-appended 2D COF (COF-TpDb) was treated with hydroxylamine (NH_2_-OH) in methanol [28] to obtain amidoxime-functionalized COF (COF-TpDb-AO) (Fig. 3a) [29]. Interestingly, COF-TpDb-AO preserved the structural regularity of the former with remarkable chemical stability and sufficient porosity intake (from 1164 to 826 m^2^/g). It was further employed as an adsorbent for uranium uptake. Remarkably, COF-TpDb-AO demonstrated superior uranium uptake in terms of saturation adsorption capacity (408 mg/g) compared to the amorphous POP-TpDb-AO sample (355 mg/g). Meanwhile, in terms of contact time, it only needs 10 or 30 min to reach 81 or 95% equilibrium adsorption capacities while POP-TpDb-AO needs 90 min to accomplish 95%. Furthermore, similar postsynthetic functionalization was reported on a newly discovered dioxin-linked 2D COF (COF-316) with remarkable crystallinity intake. Thus, it is a potential material as the solid sorbent for nuclear-waste and other metal-pollutant sequestration [30].

The thioether bond is one of the electron-rich moieties that has been successfully incorporated into COFs under a thiol-ene ‘click’ reaction as depicted in Fig. 1e [31]. A vinyl-functionalized mesoporous 2D COF (COF-V) was prepared and treated with 1,2-ethanedithiol with the assistance of azobis (isobutyronitrile) (AIBN) to afford the thioether-functionalized COF (COF-S-SH) (Fig. 3b) [32]. Sufficient crystallinity and porosity were maintained by COF-S-SH with abundant sulfur species within the channel (20.9 wt%, as thiol and thioether groups). Furthermore, it was accessed as an attractive solid sorbent for heavy-metal (such as Hg) remediation. Practically, COF-S-SH was exposed to Hg^2+^ aqueous solution (25–700 ppm) and its adsorption isotherm was collected for further analysis. Notably, COF-S-SH managed an Hg^2+^ uptake capacity as high as 1350 mg/g, which surpassed the performance of all previously reported thiol- and/or thioether-functionalized materials [33–37]. In addition, it remained crystalline after four consecutive adsorption cycles and could effectively remove Hg^2+^ with high selectivity even in the presence of a high concentration of background metal ions (Ca^2+^, Zn^2+^, Mg^2+^ and Na^+^). Inspired by this work, the hydrophobic property was successfully integrated into a similar COF scaffold to produce superhydrophobic and water-repellent mesoporous 2D COF (COF-VF) [38]. The COF-VF was obtained by treating COF-V with 1H,1H,2H,2H-perfluorodecanethiol under a common thiol-ene ‘click’ reaction. A test of the water-contact angle revealed that COF-VF was a superhydrophobic material with a static water-contact angle of 167°, while COF-V and alkyl-modified COF-V exhibited contact angles of only 113° and 122°, respectively. In addition, it was used as a hydrophobic coating agent for melamine foam, paper and magnetic liquid in which the resultant coated materials demonstrated hydrophobic properties towards all aqueous liquids including inorganic acidic and basic solutions. Besides a click reaction, the inverse-vulcanization method can also be employed to functionalize COFs via a thioether bond. For instance, the synthesis of electroactive S-COF-V with remarkable electrochemical performance was executed under post-

synthetic functionalization by involving a thioether bond [39]. In addition, a thioether-functionalized 3D COF (COF-102-SPr) has also been constructed under a thiol-ene click reaction with preserved crystallinity relative to its parent COF [40].

**Figure 4.**
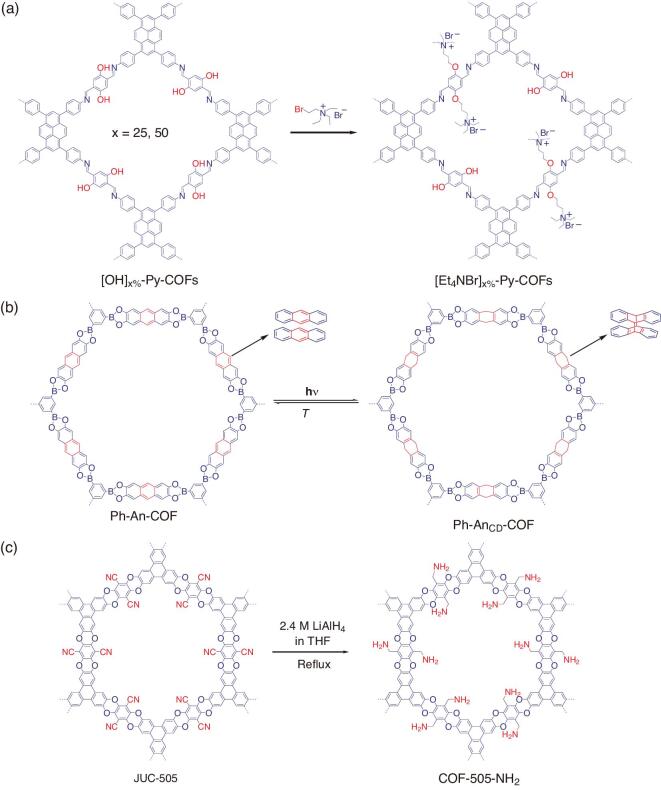
Postsynthetic functionalization of pendant groups of COFs via covalent bond, cycloaddition reaction and reduction reaction. (a) Functionalization of hydroxyl-containing [HO]_x%_-Py-COFs into ether-functionalized [Et_4_NBr]_x%_-Py-COFs via ether bond. (b) Structural transformation of Ph-An-COF into Ph-An_CD_-COF via 4π + 4π cycloaddition reaction. (c) Functionalization of nitrile-appended JUC-505 COF into amine-functionalized JUC-505-NH_2_ via reduction reaction.

The hydroxyl pendant group is one of the versatile functional groups for the functionalization of COFs due to its reactivity to several constituents. For example, a coupling reaction between hydroxyl with isothiocyanate groups affords an extended covalent thiocarbamate bond (Fig. 1f). A fluorescent active COF (T-COF-OFITC) was prepared under a coupling reaction between hydroxyl-appended large-pore T-COF-OH with a fluorescent active fluorescein-isothiocyanate (FITC) molecule via a thiocarbamate bond (Fig. 3c) [41]. Notably, structural preservation was clearly observed under powder X-Ray diffraction (PXRD) analysis and a significant surface-area reduction was found under porosity analysis. In addition, COF-5 was also functionalized under a similar route to obtain COF-5-FITC. As predicted, both functionalized COFs showed strong fluorescent signals, indicating the successful transformation of photo-inactive COFs into photoactive COFs. On another attempt, hydroxyl groups within the backbone of the COF were further condensed with electrophile leaving groups such as halide under Williamson ether reactions to obtain ether-functionalized COFs (Fig. 2g) [42]. Hydroxyl-containing mesoporous 2D imine-linked COF series ([HO]_x%_-Py-COFs) were prepared and employed as scaffolds for immobilizing catalytically active ionic liquid (IL) molecules to obtain IL-COF systems [43]. The immobilization of (2-bromoethyl)triethylammonium bromide (Et_4_NBr) on the pore wall of [HO]_x%_-Py-COFs afforded [Et_4_NBr]_x%_-Py-COFs (x = 25–100) (Fig. 4a). Notably, the structural order was seen on [Et_4_NBr]_x%_-Py-COFs relative to their pristine scaffold when x = 25 or 50 and crystallinity disturbance occurred once x reached 75 and 100 along with severe porosity reduction. Under CO_2_-adsorption analysis, [Et_4_NBr]_x%_-Py-COFs (x = 25 or 50) exhibited elevated CO_2_-uptake capacities and higher isosteric heat absorption (*Q_st_*) than those [HO]_x%_-Py-COFs samples and was able to catalyse the transformation of CO_2_ into value-added chemicals (*N*-formylation reaction). For instance, [Et_4_NBr]_50%_-Py-COF accounted for high CO_2_-uptake capacity (164.6 mg/g, at 1 bar and 273 K), which was the highest value compared to several reported COFs. Meanwhile, with 5 mol% [Et_4_NBr]_50%_-Py-COF, a solely formylated product of N-methylaniline was obtained with an isolated yield of 94% under mild conditions, while [HO]_50%_-Py-COF could only exhibit 32% conversion under similar conditions. The generality of this postsynthetic functionalization was also demonstrated on other 2D COFs. For example, the functionalization of [OH]_x%-_TD-COFs into [BE]_x%-_TD-COFs transformed the neutral skeleton of the COF scaffold into zwitterionic-functionalized COF and the design of ionic-based [SO_3_^−^]-DhaTab COF was obtained by reacting DhaTab COF with 1,3-propanesulton in toluene [44,45].

Functionalization of the structural skeleton of COFs via a covalent bond can also be facilitated by a cycloaddition reaction. Meanwhile, anthracene is a typical unit that can be transformed via a (4π + 4π) or (4π + 2π) cycloaddition reaction [46,47]. Thus, COFs with anthracene units could demonstrate this type of functionalization. Structural transformation of planar 2D sheet COF (Ph-An-COF) into a concavo-convex polygon skeleton COF (Ph-An_CD_-COF) was performed by irradiating the COF scaffold with Ar (360 nm using a Xenon lamp completed with a band-path filter) (Fig. 4b) [48]. Under this treatment, the Ph-An-COF faced a thermal reversible photo-induced (4π + 4π) cycloaddition reaction that stimulated a drastic conformational change and caused a shortened distance between the central portions of the dimeric molecules. Electronic adsorptions of both materials were essentially different due to the structural transformation while preserving their crystallinities and insignificant porosity change. This phenomenon may promise interesting applications in the field of optics and sensing. Similar structural transformation could also be initiated by using a chemical initiator rather than light irradiation. A predesigned COF scaffold (DaTp) was subjected to a [4π + 2π] Diels-Alder cycloaddition reaction with *N*-hexylmaleimide molecules, leading to the disturbance of π–π stacking interactions and planarity of the individual layers of DaTp. As a result, layer exfoliation occurred, resulting in thin-layer covalent organic nanosheet formation (DaTp-CON) [49]. Meanwhile, the pendant groups of COFs could also be functionalized or transformed via the reduction or oxidation reaction. For example, highly stable polyarylether COFs (PAE-COFs, JUC-505) bearing nitrile (-CN) pendant groups was successfully reduced to give amine-functionalized COF (JUC-505-NH_2_) (Fig. 4c) [50]. The reduction reaction was performed by refluxing JUC-505 with LiAlH_4_ in tetrahydrofuran (THF). Significantly, JUC-505-NH_2_ could effectively evacuate the tetracycline antibiotics compared to the JUC-505 event at pH = 13. In other sophisticated work, nitro-containing 2D COF (TpBD(NO_2_)_2_) was reduced (with SnCl_2_ under reflux) to produce amine-containing COF (TpBD(NH_2_)_2_ and azide-containing COF (X%[N_3_]-COFs) was also reduced (treated with PPh_3_ in methanol) to give the corresponding amine-containing COF (X%[NH_2_]-COFs) [25,51].

#### Ionic/coordination bond

Unlike the covalent bond that was formed through sharing electrons, coordination-bond formation involves the donor–acceptor electron transfer between the active sites of the components [52].

Meanwhile, ionic interaction occurs on the dipolar region between the two active sites, establishing engagement forces between the components [53,54]. These two concepts were then adopted in functionalizing COFs with metal ions or molecular ions. Based on the components involved, this structural functionalization is categorized into two following sections, namely metal ion/COF hybrid and molecular ion/COF hybrid.

**Figure 5.**
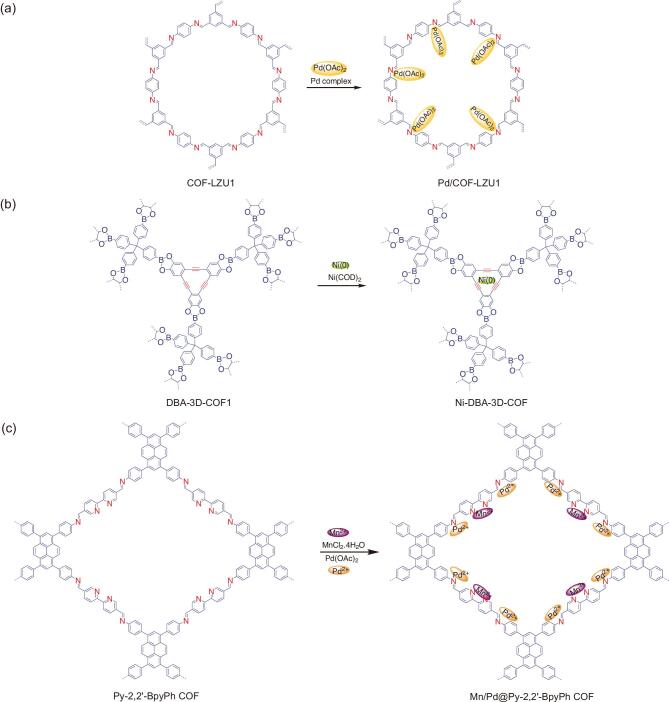
Postsynthetic functionalization of COFs via coordination bond. (a) Hybridization of Pd(OAc)_2_ into the channel of COF-LZU1 to obtain Pd/COF-LZU1. (b) Hybridization of Ni(COD)_2_ into the channel of DBA-3D-COF1 to obtain Ni-DBA-3D-COF. (c) Docking bimetal salts of Mn/Pd into the channel of Py-2,2^′^-BPyPh to obtain Mn/Pd@Py-2,2^′^-BPyPh.

##### Metal ion/COF hybrid.

COFs with electron-rich *s-* or *p*-type orbitals may facilitate the formation of coordination bonds with metals (with electron-less *d* orbitals). For example, imine linkages found in imine-linked COFs are able to govern coordination bonds with metals or metal ions [55]. More importantly, the electron-rich nitrogen species of imine linkages within 2D COFs are relatively close to each other (approximately 4 Å, the distance from sheet to sheet), thus they promote the efficient establishment of coordination bonds.

The Pd-complex, Pd(OAc)_2_, was successfully hybridized in between the adjacent layers of a microporous 2D imine-linked COF (COF-LZU1)

under simple overnight stirring in dichloromethane to afford Pd/COF-LZU1 (Fig. 5a) [56]. It was assumed that Pd species were coordinatively bonded on nitrogen atoms between the adjacent sheets. The transmission electron microscopy (TEM) images of Pd/COF-LZU1 exhibited black dots of Pd species, confirming the homogeneous dispersion of Pd species within the skeleton of COF-LZU1 while preserving the crystalline nature of the host. Remarkably, the Pd/COF-LZU1 showed an excellent catalytic activity towards the Suzuki–Miyaura coupling reaction with a broad scope of reactants. This catalytic performance outperformed Pd-containing MOF analogs under similar catalytic conditions [57]. Using a similar approach, Pd(OAc)_2_ was also hybridized in the channel of a porphyrin-containing 2D COF (H_2_P-Bph-COF) to generate Pd/H_2_P-Bph-COF with enhanced catalytic performance towards a typical Suzuki-coupling reaction [58]. Meanwhile, by using Co(OAc)_2_, the Co-TpBpy was produced by soaking and stirring the bipyridine-containing TpBpy with Co(OAc)_2_ precursor in methanol [59]. However, the hybridization of Co species in this work is coordinatively bonded in-plane with bipyridine moieties rather than in between the adjacent imine linkages of the COF. Notably, the resultant Co-TpBpy possessed enhanced electrochemical activity (oxygen evolution reaction, OER) compared to the parent COF. In a similar effort, bipyridine-containing COF was used to coordinatively bond a noble metal salt (Re(CO)_5_Cl) to generate Re-COF [60]. The resultant hybrid was employed as a photocatalyst for CO_2_ reduction into CO under visible-light illumination with excellent selectivity (98%) and much better activity compared to its homogeneous Re counterpart.

Hybridization of metal complex within the 3D COFs comes with a different pathway. 3D COFs possess a 3D pore architecture with a typical longer layer-to-layer distance (approximately 6.6 Å), which is not sufficient to perform coordination spatial cooperation between the adjacent layers. A 3D COF with a high surface area and low density (DBA-3D-COF 1) was prepared and further stirred in toluene containing 10 wt% of Ni(COD)_2_ at room temperature to afford Ni-DBA-3D-COF (Fig. 5b) [61]. The Ni species (10.1 wt%) were coordinatively bonded exclusively on the cavity of π-conjugated DBA [62] rather than on boronate ester. Interestingly, the structural order was clearly preserved and minimal surface-area reduction was observed. In the end, the Ni-DBA-3D-COF was employed as a solid sorbent for ethane/ethylene separation. On another occasion, Pd(OAc)_2_ salt was also successfully impregnated into the pore of 3D COF (COF-300) in which the Pd ions were assumed to be coordinatively bonded on solely nitrogen of a particular imine linkage within the skeleton [63]. The X-ray photoelectron spectroscopy (XPS) measurement revealed that Pd species were coordinatively bonded with imine nitrogen atoms at a 1:1 ratio; thus, they largely occupied the pore surface of the COF. The resultant Pd(OAc)_2_@COF-300 was further employed as a catalyst for a Suzuki–Miyaura coupling reaction. Notably, Pd(OAc)_2_@COF-300 showed an excellent catalytic activity (barely 99% conversion) toward a phosphine-free Suzuki–Miyaura coupling reaction as well as Heck and Sonogashira reactions. Besides imine and bipyridine moieties, the *N,N^′^*-bis [salicylidene] ethylenediamine (Salen) has been considered as an attractive moiety for coordination chemistry due to its ability to hold metal ions [64]. In line with this, a 2D Salen-COF with a large pore size was constructed and impregnated with metal salts (M = Cu, Ni, Zn, Co or Mn) under stirring to afford M/Salen-COFs [65]. This treatment did not sacrifice the crystalline nature of the former COF, since the metal ions occupied most of the Salen pockets. Since these metallosalen-COFs were highly porous and chemically stable, Co/Salen-COF was employed as a heterogeneous catalyst for Henry reactions and exhibited a good performance. Very recently, Fang *et al.* developed a microporous 3D-Salphen-COF series (JUC-508 and JUC-509) and used them as a perfect scaffold for metalation [66]. The JUC-509 was further metalated via wet chemistry by immersing it in a methanol solution of corresponding metal salts, affording the JUC-509-Y series (Y = Mn, Cu or Eu). They were further employed as heterogeneous catalysts for the catalytic removal of superoxide radical anion (O_2_^.-^).

The incorporation of a multi-metal component into the pore of COFs has also been demonstrated. The successful docking of bimetallic salts of Mn/Pd into the pore of a dual-coordinative site containing Py-2,2^′^-BPyPh was performed via sequential impregnation treatment, where MnCl_2_ was previously impregnated to give Mn@Py-2,2^′^-BPyPh and followed by treatment with Pd(OAc)_2_ to afford bimetal Mn/Pd@Py-2,2^′^-BPyPh (Fig. 5c) [67]. Based on ICP-OES analysis, the content of the Mn species was 0.8 wt% while the content of the Pd species was 9.3 wt%. The Mn species could only coordinate on the bipyridine moieties and the Pd species docked at the imine sites. Interestingly, the resultant hybrid was able to catalyse a Heck-epoxidation tandem reaction. Notably, Mn/Pd@Py-2,2^′^-BPyPh performed 94% conversion to achieve the final product with the stepwise reaction giving 95 and 98% conversion for the heck and epoxidation reactions, respectively. By using the similar COF as a scaffold, two metallic salts of Rh and Pd have successfully been docked on the pore surface of Bpy-COF to obtain another bimetallic docking COF hybrid (Rh/Pd@X%Bpy-COF) with the ability to catalyse a one-pot addition–oxidation reaction [68]. Furthermore, another strategy was carried out by preparing dual imine and carboxyl coordinative sites containing COF([HOOC]_X_-COFs, x = 17, 33, 50 or 100) to hybridize a multi-metal component [69]. Various metal ions (Ca^2+^, Mn^2+^ and Sr^2+^) were then integrated into the [HOOC]_17_-COF by simply mixing the COF with the chloride salt solution of the respective metals and afforded [MOOC]_17_-COFs (M = Ca^2+^, Mn^2+^ or Sr^2+^). As predicted, the resultant hybrids exhibited enhanced ammonia uptakes relative to the bare COF.

**Figure 6.**
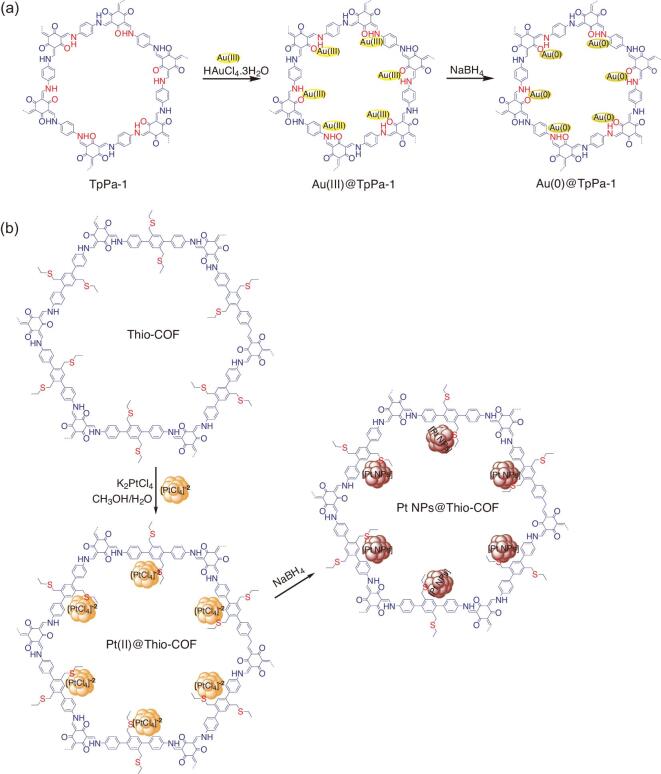
Postsynthetic functionalization of COFs via coordination bond. (a) Deposition of Au nanoparticle into the channel of imine-linked TpPa-1 to obtain Au(0)@TpPa-1. (b) Deposition of Pt metal cluster into the channel of Thio-COF to obtain PtNPs@COF.

Similar to the metal ion/COF hybrid, the deposition of metal nanoparticle species within the pore of COFs has also been reported. Typically, the hybridized metal ions within the COF channel are further reduced into either a single nanoparticle or bulky metals to produce M@COF and/or M_x_O_y_@COF hybrids. The Au nanoparticles (Au(0)) were deposited within the channel of imine-linked TpPa-1 by reducing (using NaBH_4_ solution) the deposited Au(III) ions in Au(III)@TpPa-1 to give Au(0)@TpPa-1 (Fig. 6a) [70]. The resultant Au(0)@TpPa-1 was used as a high-performance catalyst for reducing 4-nitrophenol to 4-aminophenol. As predicted, Au(0)@TpPa-1 performed an excellent catalytic activity compared to the pristine COF. For instance, 100% reactant conversion was achieved in only 13 min for Au(0)@TpPa-1 (1.20 wt%), while it required 20 min for the Au salt (HAuCl_4_·3H_2_O) and no catalytic activity was observed for the COF-TpPa-1 sample. Meanwhile, the Pd(0)@TpPa-1 has also been obtained under similar procedures and further employed as a catalyst for both C–C Sonogashira/Heck coupling and C–H activation reactions [71–74]. Furthermore, since Pd metal is a highly active catalyst, several Pd(0)@COF hybrids have been produced and utilized as catalysts so far [75,76].

**Figure 7.**
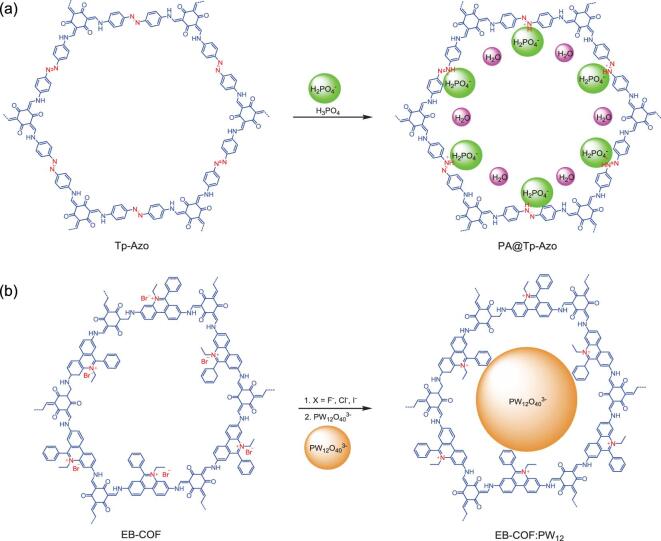
Postsynthetic functionalization of COFs via ionic bond. (a) Hybridization of phosphoric acid (PA) into the channel Tp-Azo COF to obtain PA@Tp-Azo. (b) Hybridization of polyoxometalate (PW_12_) into the channel of cationic EB-COF:Br to obtain EB-COF: PW_12_.

On the other hand, besides imine-linked COFs, other types COFs that have coordinative sites have potential as scaffolds. For example, a hydrazone-linked COF (COF-ASB) was designed and used as a scaffold to accommodate Ru nanoparticles to give Ru@COF-ASB [77]. The deposition of Ru nanoparticles was done by mixing COF-ASB with a CH_3_CN solution of ruthenium chloride (RuCl_3_) and further reduced by NaBH_4_. Notably, the hybrid showed enhanced catalytic performance for a one-pot solvent-free tandem reaction of imine synthesis from alcohols [78] and also in an oxidation reaction relative to the bare COF. Deposition of a metal cluster into the channel of a COF has also been done. An electron-rich thioether-containing COF (Thio-COF) was designed that was able to strongly hold the metal species and control metal nanoparticle nucleation [12,79]. The metalation was performed by mixing Thio-COF suspension with K_2_PtCl_4_ solution, which was then reduced with NaBH_4_ in methanol to afford PtNPs@COF (Fig 6b). Interestingly, under TEM exploration, it was found that the Pt species were obtained as crystalline ultra-small and uniform nanoparticles with an average size of 1.7 nm. The PdNPs@COF analog was also synthesized via a similar route by using K_2_PdCl_4_ as the metal precursor. Both hybrids were then employed as catalysts for reducing 4-nitrophenol to 4-aminophenol and Suzuki–Miyaura coupling reactions. Both catalysts presented excellent catalytic performances that outperformed the unsupported Pt nanoparticles (NPs) catalyst. On the other hand, the deposition of metals with two different oxidation states has also been reported, revealing robust postsynthetic metal hybridization in COFs via a coordination bond [80].

**Figure 8.**
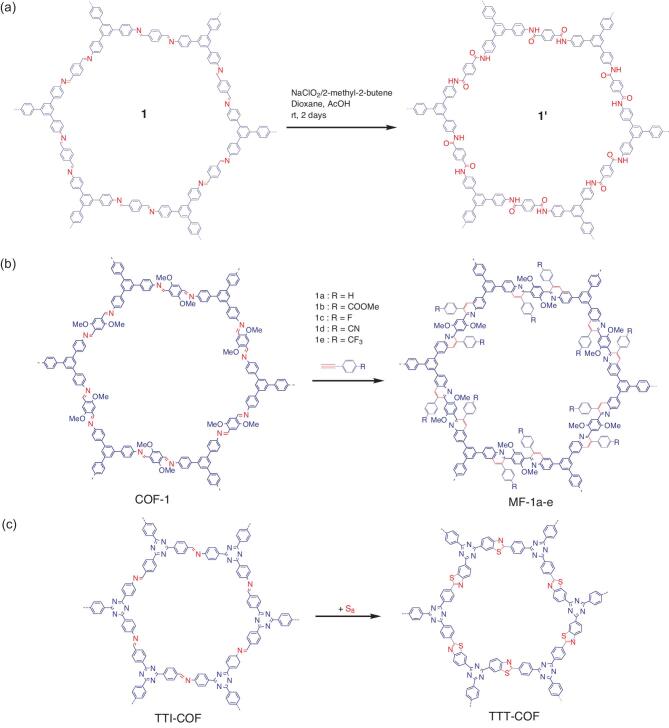
Postsynthetic functionalization of linkage of COFs. (a) Linkage transformation of imine-linked TPB-TP-COF(**1**) into amide-linked COF (**1^′^**) under oxidation reaction. (b) Linkage transformation of imine-linked COF-1 into quinone-linked COFs (MF-1a-e) under aza-Diels-Alder cycloaddition reaction. (c) Linkage transformation of imine-linked TTI-COF into thiazole-linked COF (TTT-COF).

##### Molecular ion/COF hybrid.

The coordinative and ionic sites within the skeleton of COFs can also engage the deposition of molecular ions via ionic interaction. To this end, typical ionic liquids, inorganic/organic salts and other molecular ions have been incorporated within the channel of COFs—for example, the deposition of phosphoric acid (PA, H_2_PO_4_^−^) into the channel of a highly stable 2D COF (TP-Azo) to afford PA@Tp-Azo (Fig. 7a) [81]. The azo moieties within the COF could stabilize H_2_PO_4_^−^ once protonated [82]. Notably, PA@Tp-Azo reported remarkable proton conductivity as high as 9.9 × 10^−4^ S/cm at 332 K and 98% relative humidity (RH)—much higher than PA@Tp-Stb. On another occasion, a molecular ion of PA was again loaded into the channel of two aza-fused COFs (aza-COF-1 and aza-COF-2) to produce aza-COF-1_H_ and aza-COF-2_H_, respectively [83]. As predicted, both hybrids showed dramatic improvement in proton conductivities relative to their parent COFs. Meanwhile, in another report, the molecular ion of polyoxometalate (POM) was loaded into the channel of a cationic COF (EB-COF:Br, Br as counter ion) [84]. Ion-exchange treatment was performed with PW_12_O_40_^3−^ (abbreviated as PW_12_) to give EB-COF:PW_12_ (Fig. 7b). Remarkably, the ion-exchange treatment did not severely alter the rigidity and crystallinity of the framework, signaling the robustness of the COF scaffold. The porosity of the obtained COF hybrid was almost diminished due to the occupation of the large molecule of POM. For instance, the surface area of EB-COF:Br was 774 m^2^ g^–1^, while EB-COF:PW_12_ showed a surface area of 8 m^2^ g^–1^. With POM molecules in the channel of EB-COF, the resultant EB-COF:PW_12_ demonstrated a proton conductivity 100 times higher than that of the EB-COF:Br sample. Meanwhile, by using an anionic [Mo_3_S_13_]^2−^ cluster, Mo_3_S_13_@EB-COF has also been fabricated and employed as a photocatalyst material [85,86]. In addition, application in lithium-ion conduction has also met with the fabricated molecular ion/COF hybrid of PEG-Li^+^@EB-COF-ClO_4_ [87]. Nevertheless, 3D COF has also been employed to accommodate molecular ions (denoted as PMA@COF-300) with enhanced performance as a catalyst [88].

### Linkage functionalization

The linkages in COFs are typically stable but somehow reactive to certain components and under particular conditions. Functionalization or transformation of the linkage of COFs has been performed so far through certain reactions, including oxidation–reduction and ring-fusion reactions. The imine linkage, which is relatively more stable than the boroxine linkage, has been widely transformed. For example, typical 2D imine-linked COF (TPB-TP-COF,1) was treated under oxidation reaction using sodium chlorite (as an oxidant), acetic acid (as a buffer) and 2-methyl-2-butene (Fig. 8a) [89,90]. This oxidation treatment transformed imine linkage into amide linkage, thus a new amide-linked COF (1^′^) was obtained. Under similar conditions, 4PE-1P-COF (2) was also oxidized into respective amide-linked COF (2^′^). Notably, the both resultant amide-linked COFs preserved the structural order and remarkable chemical stabilities relative to their pristine COFs, including in 12 M HCl and 1 M NaOH for 24 h. In another occasion, 2D (COF-366-M) and 3D (COF-300) imine-linked COFs were transformed by reducing their linkage into amine-linked COF (366-M-AR and COF-300-AR) [91]. The reduction reaction was performed by quantitatively treating the corresponding COFs with NaBH_4_. Again, the chemical stability of the resultant amine-linked COFs was somehow enhanced to a high extent relative to their pristine COFs. In addition, both 366-M-AR and COF-300-AR showed improved electrochemical activities compared to the unreduced counterparts.

Meanwhile, enhancing the structural integrity of an imine-linked COF (termed as COF-1) from moderately stable into ultra-stable quinone-linked COF (denoted as MF-1a-e) has been demonstrated by kinetically fixing its linkage via an aza-Diels-Alder cycloaddition reaction (Fig. 8b) [92]. The reaction involved the efficient Povarov (aza-DA) reaction between aryl imines and arylalkynes by subjecting the COF scaffold to phenylacetylene at 110°C in the presence of BF_3_.Et_2_O, chloranil and toluene for 72 h [93]. Drastic chemical stability was observed for all resultant MF-1a-e COFs. For instance, they were stable in strong acid (12 M HCl at 50°C, 8 h), superacid (98% TfOH, 3 days), strong base (14 M NaOH in H_2_O/MeOH at 60°C, 24 h), strong oxidant (KMnO_4_ in H_2_O/CH_3_CN, 24 h) and reducing agent (NaBH_4_ in MeOH at 65°C, 24 h). In another report, an imine-linked COF (TTI-COF) was topologically transformed by arresting its crystalline state via a reversible to irreversible ring-fused thiazole-linked COF (TTT-COF) (Fig. 8c) [94,95]. The transformation utilized elemental sulfur reacted with aromatic imine to first oxidize the imine to a thioamide and subsequently oxidatively cyclized the thioamide group to form a thiazole ring [96]. Another reported approach of transforming the linkage of COFs was a linkage-exchange method. An imine-linked COF (ILCOF-1) was treated using either with 4 equiv. 2,5-diaminobenzene-1,4-dithiol dihydrochloride in N,N-dimethylformamide (DMF)/water to

give thiazole-linked COF (COF-921) or with 2,5-diaminohydroquinone dihydrochloride in DMF under oxygen atmosphere to obtain oxazole-linked COF (LZU-192), respectively [97]. These linkage transformations were performed via consecutive steps, initialized by linker exchange, followed by cyclization and end with an oxidation reaction in a one-pot system. As predicted, both COF-921 and LZU-912 were chemically stable relative to ILCOF-1 including in basic (10 M NaOH) and acidic solutions (12.1 M HCl, 18 M H_2_SO_4_, 14.8 M H_3_PO_4_ and 9 M H_2_SO_4_ in dimethyl sulfoxide (DMSO)) for 1 day. Although the linkage transformation discussed in this section mainly utilizes imine linkage, other linkages may also possibly be functionalized via a certain reaction or treatment.

### Host–guest functionalization

As highly porous materials, COFs are perfect porous confinement materials acting as hosts to accommodate guest molecules with controlled quantities via the host–guest chemistry approach. This confinement property involves weak forces that have been well studied in general supramolecular chemistry including van der Walls, π–π interaction and hydrophobic/hydrophilic interactions [98].

**Figure 9.**
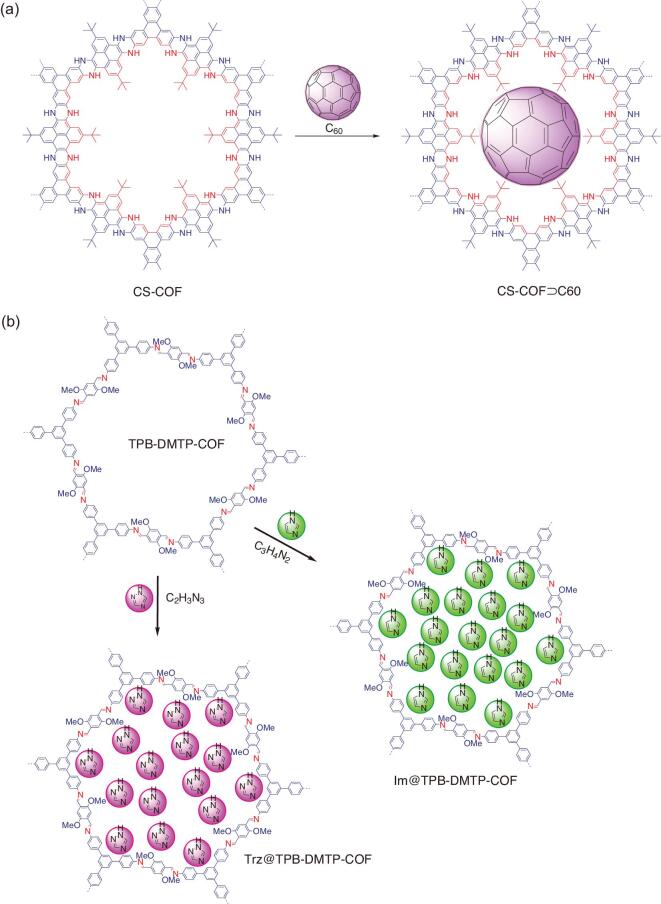
Postsynthetic host–guest functionalization of COFs. (a) Hybridization of bulk-C_60_ molecules into the pore channel of phenazine-linked CS-COF to produce CS-COF⊃C60. (b) The loading of proton carriers of triazole (trz) and imidazole (imi) into the pore channel of TPB-DMTP-COF to obtain proton conductors based trz@TPB-DMTP-COF and im@TPB-DMTP-COF.

The photo-responsive bulk-C_60_ molecules were incorporated into the channel of the highly stable, electronically conjugated 2D phenazine-linked COF (CS-COF) via host–guest chemistry to obtain donor–acceptor-based CS-COF⊃C60 [99]. The loading of bulk-C_60_ molecules was executed under a thermal sublime diffusion method (Fig. 9a). As high as 25 wt% of bulk-C_60_ molecules have been successfully loaded into the channel of the host, suggesting a peapod-like encapsulation. The resultant hybrid was then employed as on–off ratio photoswitches and photovoltaic cell. The device made by CS-COF⊃C60 (with optimized polymethyl methacrylate (PMMA) content) showed the best on–off ratio photoswitches (5.9 × 10^7^), photocurrent performance (as high as 2.0 μA) and dark current of 0.029 pA at a bias voltage of 1.5 V. In addition, it allowed multiple rounds of on–off switching without a loss of performance at room temperature that was superior to all other COFs, including hole-conducting-type COFs (8 × 10^4^ for PPy-COF [100] and 150 for NiPc-COF [101]) and electron-conducting type COFs (60 for NiPc-BTBA COF [102], 5 × 10^4^ for ambipolar-conducting ZnP-COF [103] and 1.3 × 10^4^ for 2D D-A COF [104]). In a similar vision, the electron acceptor [6,6]-phenyl-C_61_-butyric acid methyl ester (PCBM) molecules were loaded into the channel of a boronate ester-linked TT-COF to generate an electron donor–acceptor heterojunction–COF hybrid (TT-COF:PCBM) [105]. The interaction of both components was assumed to involve a van der Waals interaction. Since thieno[2,3-b]thiophene-containing building blocks exhibited high charge-carrier mobilities and efficient photo-induced charge transfer from polymerized thienothiophene derivatives to fullerene acceptor molecules [106,107], the resultant TT-COF:PCBM was an attractive candidate for organic photovoltaic material. Meanwhile, the conductivity of the redox-active DAAQ-TFP COF film was enhanced by electropolymerizing redox-active 3,4-ethylenedioxythiophene (EDOT) monomer into its pores to give PEDOT⊃DAAQ-TFP-COF [108]. As predicted, the hybrid film exhibited dramatically improved current responses in CV experiments when compared with DAAQ-TFP-COF film. Notably, it could accommodate a high charging rate without compromising performance and exhibited both a 10-fold higher current response and stable capacitance over 10 000 cycles relative to the pristine host.

Another piece of sophisticated work was loading *N*-heterocyclic proton carriers (triazole (trz) and imidazole (im) molecules) in the channel of hexagonally aligned, mesoporous and highly stable TPB-DMTP-COF to obtain trz@TPB-DMTP-COF and im@TPB-DMTP-COF as proton conductors (Fig. 9b) [109]. The loading of these proton carriers was done via the thermal-vaporization method at 150°C or 120°C overnight, and 180 wt% trz and 155 wt% im could be loaded into the host, respectively. The resultant hybrids were explored for proton conduction experiments in which trz@TPB-DMTP-COF documented a proton conductivity as high as 1.1 **×** 10^−3^ S cm^–1^ at 130°C, while im@TPB-DMTP-COF exhibited a proton conductivity of 4.37 **×** 10^−3^ S cm^–1^, which were pretty much higher compared to their pristine COF. Furthermore, a catalytically active biomolecular has also been engaged into the pore of a predesigned COF as host, exemplifying the generality of host–guest functionalization in COFs [110]. Meanwhile, 3D COF has also been employed as a host to demonstrate the host–guest confinement phenomenon. For example, the organometallic molecules of [Fe(η^5^-C_5_H_5_)_2_], [Co(η^5^-C_5_H_5_)_2_] and [Ru(cod)(cot)] were loaded into the pore of COF-102 to obtain (FeCp_2_)_4_@COF-102, (CoCp_2_)_4_@COF-102 and [Ru(cod)(cot)]@COF-102 [111]. Interestingly, the resultant hybrids showed various confinement phenomena; thus, it provided insights for further research.

## Conclusions

COFs materials with tunable structural topologies and high thermal/chemical stabilities provide a versatile postsynthetic functionalization platform to enhance their psychochemical properties and performances toward specific applications. The manageable porosities and functionalities via the bottom-up design of COFs allow judicious incorporation of constituents that can be bonded by strong covalent bonds, ionic/coordination forces and host–guest chemistry interactions. Nevertheless, their linkages can also be postsynthetically functionalized to improve their structural stability. Furthermore, the resultant functionalized COFs with particular hybrid or composite fashions show outstanding performances in the field of catalyst, gaseous uptake–release, optics, energy storage and so on. In the future development of COFs, we assume that there are still many gaps left for further explorations, especially in functionalization of the available linkages that are less studied. In addition, incorporations of functional constituents via host–guest chemistry so far have been mainly developed for electronic and energy-storage applications, thus investigations in wider sophisticated applications are still highly demanded. Last but not least, the postsynthetic functionalization of 3D COFs is still less studied; thus, continuous efforts in the development of 3D COFs may need special concern.
